# Cytoskeletal gene alterations linked to sorafenib resistance in hepatocellular carcinoma

**DOI:** 10.1186/s12957-024-03417-2

**Published:** 2024-06-07

**Authors:** Hong Xiao, Hangyu Chen, Lei Zhang, Maimaitiyasen Duolikun, Baixin Zhen, Subinuer Kuerban, Xuehui Li, Yuxi Wang, Long Chen, Jian Lin

**Affiliations:** 1 Key Laboratory of Tropical Biological Resources of Ministry of Education, School of Pharmaceutical Sciences, Hainan University, Hainan, China; 2 Department of Pharmacology, Xinjiang Medical University, Urumqi, China; 3 Department of Pharmacy, Peking University Third Hospital, 49 Huayuan North Rd, Haidian District, Beijing, 100191 China; 4 Synthetic and Functional Biomolecules Center, Beijing National Laboratory for Molecular Sciences, Peking University, 49 Huayuan North Rd, Haidian District, Beijing, 100191 China; 5 Peking University, Third Hospital Cancer Center, 49 Huayuan North Rd, Haidian District, Beijing, 100191 China

**Keywords:** KAS-seq, Hepatocellular carcinoma, Sorafenib, Drug resistance, Cytoskeleton

## Abstract

**Background:**

Although sorafenib has been consistently used as a first-line treatment for advanced hepatocellular carcinoma (HCC), most patients will develop resistance, and the mechanism of resistance to sorafenib needs further study.

**Methods:**

Using KAS-seq technology, we obtained the ssDNA profiles within the whole genome range of SMMC-7721 cells treated with sorafenib for differential analysis. We then intersected the differential genes obtained from the analysis of hepatocellular carcinoma patients in GSE109211 who were ineffective and effective with sorafenib treatment, constructed a PPI network, and obtained hub genes. We then analyzed the relationship between the expression of these genes and the prognosis of hepatocellular carcinoma patients.

**Results:**

In this study, we identified 7 hub ERGs (ACTB, CFL1, ACTG1, ACTN1, WDR1, TAGLN2, HSPA8) related to drug resistance, and these genes are associated with the cytoskeleton.

**Conclusions:**

The cytoskeleton is associated with sorafenib resistance in hepatocellular carcinoma. Using KAS-seq to analyze the early changes in tumor cells treated with drugs is feasible for studying the drug resistance of tumors, which provides reference significance for future research.

**Supplementary Information:**

The online version contains supplementary material available at 10.1186/s12957-024-03417-2.

## Introduction

Since 2007, sorafenib, an orally administered multiple-target tyrosine kinase inhibitor (TKI), has been consistently used as a first-line treatment for advanced hepatocellular carcinoma (HCC) [1]. The anti-tumor activity of sorafenib is largely ascribed to suppressing tumor cell proliferation, inhibiting antiangiogenic activity, and promoting apoptosis [2–7]. However, only a small number of patients can benefit from sorafenib, and this population usually acquires drug resistance within 6 months [8]. Several mechanisms of resistance to sorafenib are reported, such as loops of the phosphatidylinositol-3-kinase (PI3K)/protein kinase B (Akt) and the Janus kinase (JAK)-signal transducer and activator of transcription (STAT) pathway, the hypoxic microenvironment, epithelial-mesenchymal transition or transformation (EMT), cancer stem cells, or disabling of pro-apoptotic signals [9–12]. In addition, recent studies have demonstrated that cytoskeletal proteins may be associated with drug resistance in HCC [13, 14]. However, the mechanism of sorafenib resistance in HCC still needs further study.

Tumor resistance is closely related to single-stranded DNA (ssDNA) [15–18]. Nearly all cellular processes involving the genome, such as transcription, DNA replication, DNA repair recombination, and R-loops, result in the formation of ssDNA [19–23]. This implies that ssDNA is closely related to the fate of tumor cells. During cancer progression, genetic and epigenetic alterations, microenvironment changes, and/or treatment-imposed selective pressures result in changes in tumor cells undergoing molecular and phenotypic alterations, thereby contributing to tumor heterogeneity and therapy resistance [24]. Thus, the early changes in tumor cells after in vitro drug treatment simulating tumor drug therapy may lead to the plasticity of tumor cells, thereby promoting tumor resistance.

RPA chromatin immunoprecipitation sequencing (RPA-seq), RAD51 chromatin immunoprecipitation sequencing (RAD51-seq), single-stranded DNA sequencing (SSDS), ssDNA-associated protein Rad52 ChIP-seq, and SPO11-oligo-seq, are available for the sequencing and analysis of ssDNA [25–30]. However, these approaches are based on chromatin immunoprecipitation sequencing (ChIP-seq) of single-stranded DNA binding protein complex (RPA) or particular ssDNA-associated proteins (RAD51, DMC1, SPO11 and Rad52) [25, 28, 30]. Therefore, these methods cannot reflect ssDNA profiles of the entire genome, which limits their application. Recently, it has been reported that N_3_-kethoxal-assisted ssDNA sequencing (KAS-seq) is capable of mapping all ssDNA regions across the whole genome [31, 32]. This method, based on the click chemical reaction between N_3_-kethoxal and exposed amine groups on guanine in ssDNA, can efficiently capture genome-wide ssDNA and be enriched through affinity pull-down. Moreover, KAS-seq can directly reflect the activity of RNA polymerase and is capable of detecting the dynamic changes of active transcription [31]. This suggests that the distribution of KAS-seq signals can represent the degree of double-stranded DNA opening, as well as active transcriptional extension.

In our study, we used KAS-seq technology to analyze hepatocellular carcinoma cell that were treated with sorafenib. Our results demonstrated that cytoskeleton-related genes were associated with sorafenib resistance in hepatocellular carcinoma.

## Materials and methods

### Cell culture and cell viability assay

Human liver cancer cell lines (SMMC-7721) were obtained from SHANGHAI AOLU BIOLOGICAL TECHNOLOGY CO., LTD (Shanghai, China). The cells were grown in 1640 with 10% FBS (Gibco, California, USA), antibiotics penicillin (100 U/mL) and streptomycin (100 μg/mL) at 37℃ under 5% CO_2_.

For quantification of drug response, we used the normalized growth rate inhibition (GR) of the drug treatment on SMMC-7721 cells for 24 h as the drug treatment concentration for subsequent cell sequencing samples. The GR value is:

$$GR\left(c\right)=2^\frac{{\text{log}}_2\left(x\left(c\right)/x_0\right)}{{\text{log}}_2\left(x_{ctrl}/x_0\right)}-1$$

where *x(c)* and *x*_*ctrl*_ are as described above, and *x*_*0*_ is the 50%-trimmed mean of the cell count from a sample grown in parallel and measured just prior to drug exposure.

The GR value indicated partial growth inhibition (when it lies between 0 and 1), complete cytostasis (when it equals 0) or cell death (when it lies between 0 and − 1). Compared to traditional indicators, the GR value is more robust when assessing the impact of drugs on cell signaling and growth [33].

To determine the GR50, the net A450nm determined by the CCK-8 method was represented the number of live cells in sorafenib. We inoculated the cells onto a 96-well plate and cultured them in an incubator at 37 °C with 5% CO_2_. When the cell confluence reached approximately 70%, we changed the medium and added sorafenib (Macklin, Shanghai, China) at different concentrations for 24 h of treatment, followed by 2 h of treatment with the CCK8 reagent (MCE, New Jersey, USA). Finally, colorimetric determination was carried out, and the A450 value was read under a wavelength of 450 nm using an enzyme-linked immunosorbent assay reader. Each experiment was repeated three times. Online GRcalculator tools (http://www.grcalculator.org) were then employed for calculation, analysis and visualization of dose–response data using GR approach [34].

### Cell treatment and KAS-seq

In the experimental design, the control group consists of samples that have not been treated with sorafenib, but have been supplemented with an equivalent volume of DMSO. The experimental group consists of samples that have been treated with sorafenib for specific durations (15 min, 30 min, 1 h, 2 h). Furthermore, each group has one technical replicate, and the experiment was repeated four times. Subsequently, we diluted a 100 μM stock solution of sorafenib in complete 1640 medium to the desired concentration. The SMMC-7721 cells were then treated with the diluted medium containing sorafenib (8.35 μM) for 15 min, 30 min, 1 h, and 2 h, respectively. As a control, an equal volume of DMSO was added to the control group. Additionally, we prepared a 5 mM solution of N3-kethoxal, and co-incubated it with the cells for 10 min to facilitate the labeling of genomic single-stranded DNA (ssDNA). Cell suspensions were then transferred to 1.5 ml Centrifuge tubes and 1500 rpm for 3 min. We next discarded the supernatant medium and extracted genomic DNA using Quick-DNA™ Miniprep Plus Kit (ZYMO, CA, USA).

We then referred to the established KAS-seq protocol, and ssDNA with N3-kethoxal label was biotinylated, enriched, and fragmented [32]. Dual index libraries were constructed for high throughput sequencing using xGen™ Methyl-seq Lib Prep 96rxn (IDT, CA, USA), and KAS-seq was performed on Illumina NovaSeq 6000 platform.

### Data collection

In this study, 150-bp paired-end reads were generated on the Illumina NovaSeq 6000 platform (sequenced by Annoroad). The raw reads were trimmed using the trim-galore package (v0.6.10) under default parameters, and then aligned to the human reference genome (hg19) using bowtie2 (v 2.2.9). SAM tools (version 1.9) (parameters used: Sam tools view-f 2-F 1548 -q 30) were used to filter the reads. We then converted the paired-end reads into the Bed Graph format and normalized them to the total quantity of aligned reads using bed tools (version 2.19.1). Simultaneously, we also converted the paired-end reads into the BigWig format using UCSC bedGraphToBigWig for visualization assisted by the Integrated Genomics Viewer. MACS2 (version 2.1.1) was used for peak calling to identify potential ssDNA enriched regions. Finally, we annotated the ssDNA enriched regions using the CHIP seeker package (v 1.38.0), and the genes closest to these regions were used for subsequent analysis.

The GSE109211 dataset, containing data of 140 tumor samples with clinical information, including treatment and outcome, was then selected from the Gene Expression Omnibus (GEO) database. We selected patients treated with sorafenib from this dataset and grouped them based on the effectiveness of the treatment (with the effective group serving as the control group) for differential genes analysis. In addition, 20 genes associated with the hub genes identified from the analysis were downloaded from GeneMANIA, which is real-time multiple association network integration algorithm for predicting gene function [35].

### Mapping and identifying KAS-seq signal distribution at different time points

By aligning KAS-seq data to the human reference genome (hg19), we generated a signal distribution profile for single-stranded DNA (ssDNA). This step aligned the sequenced DNA fragments to their original genomic locations, allowing us to see where the ssDNA is located in the genome.

### Analysis of differentially expressed genes

Based on the signal distribution of ssDNA from KAS-seq data, we selected the time point with the highest KAS-seq signal (1 h) for differential analysis. According to the employed filtering criteria (|log_2_FC|> 1 and *P*-value of < 0.05), differentially expressed genes (DEGs) in the one-hour and 0 min sorafenib-treated cells were analyzed using the “DEseq2” package in R. In addition, differential analysis was performed between the 46 non-responder tumor samples and 21 responder tumor samples of sorafenib treatment using the “limma” package. The data were then considered statistically significant if |log_2_FC > 1| and *P*-value < 0.05 for the differentially expressed genes (efficacy-related genes) (ERGs). We then intersected upregulated DEGs with upregulated ERGs or downregulated DEGs with downregulated ERGs to obtain early-changing genes related to efficacy.

### GO function and KEGG pathway enrichment analysis

In this study, for DEGs, ERGs and GeneMANIA-predicted genes, Gene ontology (GO) enrichment and Kyoto Encyclopedia of Genes and Genomes (KEGG) pathway analysis were than performed using the DAVID database [36]. The *P*-value < 0.05 was statistically significant and was the thresholds for selecting the major enrichment functions and pathways of DEGs, ERGs and GeneMANIA-predicted genes.

### PPI network construction and identification of hub genes

Search Tool for the Retrieval of Interacting Genes/Proteins (STRING) is a database of known and predicted protein–protein interactions [37].

After intersecting the upregulated and downregulated genes (DEGs and ERGs), we consolidated the overlapping genes. Subsequently, we imported this curated gene set into STRING, setting a minimum required interaction score (confidence level > 0.4). We then downloaded the protein–protein interaction (PPI) network in TSV format. Using Cytoscape software, we visualized the PPI network from the TSV file and employed the cytoHubba plugin to identify key nodes and subnetworks within the network. By applying the Maximum Clique Centrality (MCC) topological analysis algorithm in cytoHubba, we identified the top 10 crucial genes within the PPI network [38].

### UALCAN and human protein atlas database analysis

The University of Alabama at Birmingham cancer data analysis (UALCAN) portal is a comprehensive, user-friendly, and interactive web resource for analyzing cancer OMICS data [39]. The database was used to evaluate the expression levels of 10 hub genes between the tumor samples and normal samples. LIHC patient survival was then analyzed to evaluated clinical implications the hub genes. The Human Protein Atlas (HPA) is a comprehensive database of human proteins, with its primary objective being to provide a detailed description of the expression patterns of human genes and proteins. Protein expression data for 7 hub genes was obtained from the HPA database.

### Gene set enrichment analysis

Gene Set Enrichment Analysis (GSEA) is utilized to evaluate the distribution trend of genes in a pre-defined gene set within a gene list sorted by phenotype relevance, thereby determining their contribution to the phenotype [40]. The selected RNA-seq data from the GEO database were downloaded and subjected to GSEA for 7 hub genes. The c2.cp.kegg.v7.4.symbols.gmt subset was then downloaded from the Molecular Signatures Database to evaluate related pathways and molecular mechanisms [41]. Based on the gene expression profile and phenotype grouping, the minimum gene set was then set to 5, the maximum gene set to 5000. After performing the permutation test 1,000 times, gene sets generating a *P*-value of < 0.05 were considered statistically significant.

### Statistical analysis

Differences between the two groups were compared using Student’s t-tests, and the results were expressed as means ± standard deviations. *P*-value of < 0.05 was considered the threshold for statistical significance.

## Results

### Characterization of single-stranded DNA (ssDNA) signal distribution in KAS-seq

**Fig. 1:**
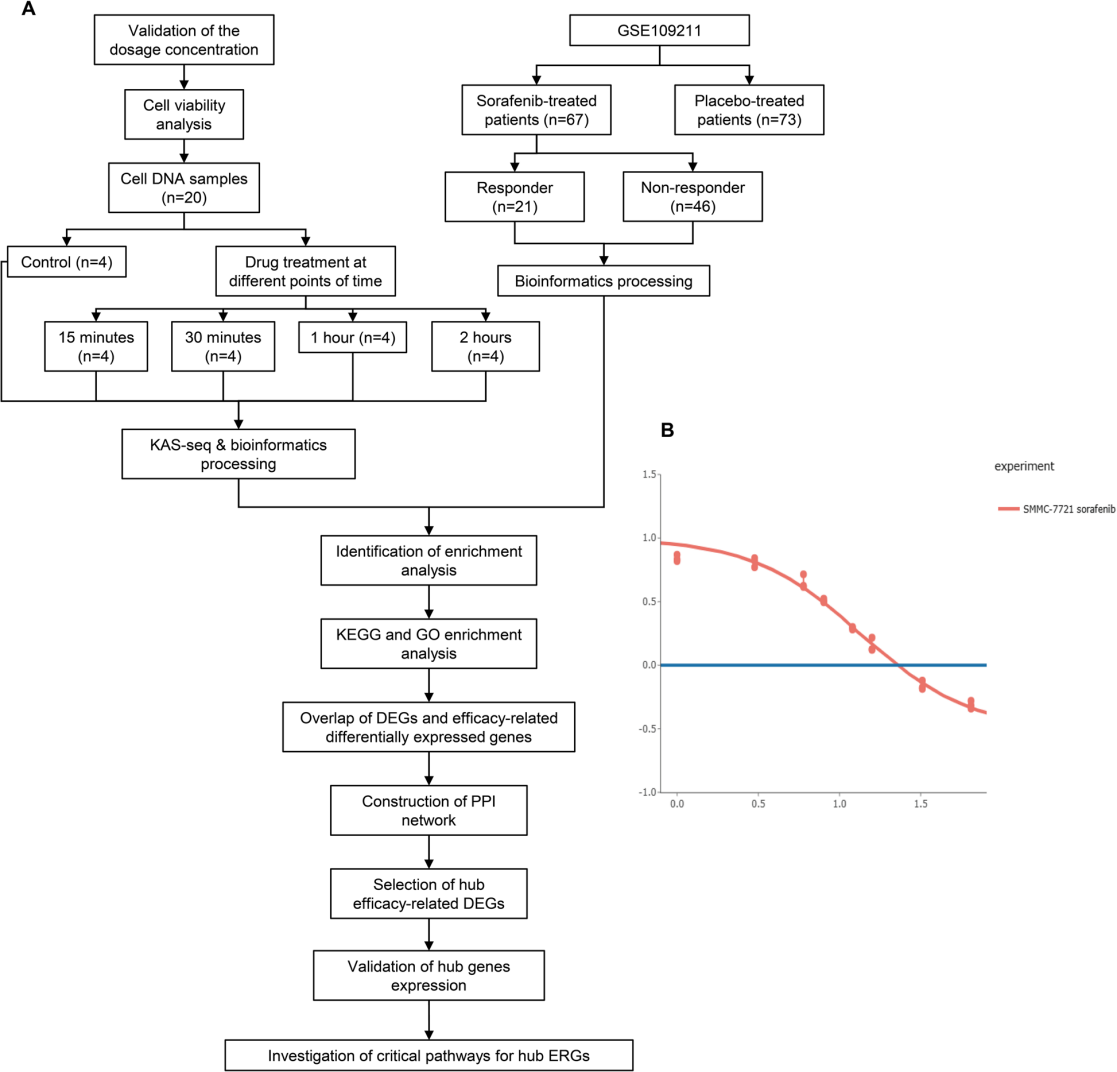
**A** Overview of study design. **B** Effect of treatment for 24 h with sorafenib on cell proliferation and viability as determined by the CCK-8 assay. Graphs show the effect of various sorafenib concentrations (*x*-axis, logarithmic values) on relative cell viability (*y*-axis, net A450 nm using CCK-8 assay). Under sorafenib treatment *versus* sorafenib-untreated control. Sorafenib concentrations were 1, 3, 6, 8, 12, 16, 32, 64 μM. Graph was obtained from the online tool GR calculator (www.grcalculator.org)

The flowchart delineates the central concept of this study (Fig. 1A). Using the Online GRcalculator tools, we analyzed and found that the concentration of sorafenib at GR50 is 8.35 μM (Fig. 1B).

**Fig. 2:**
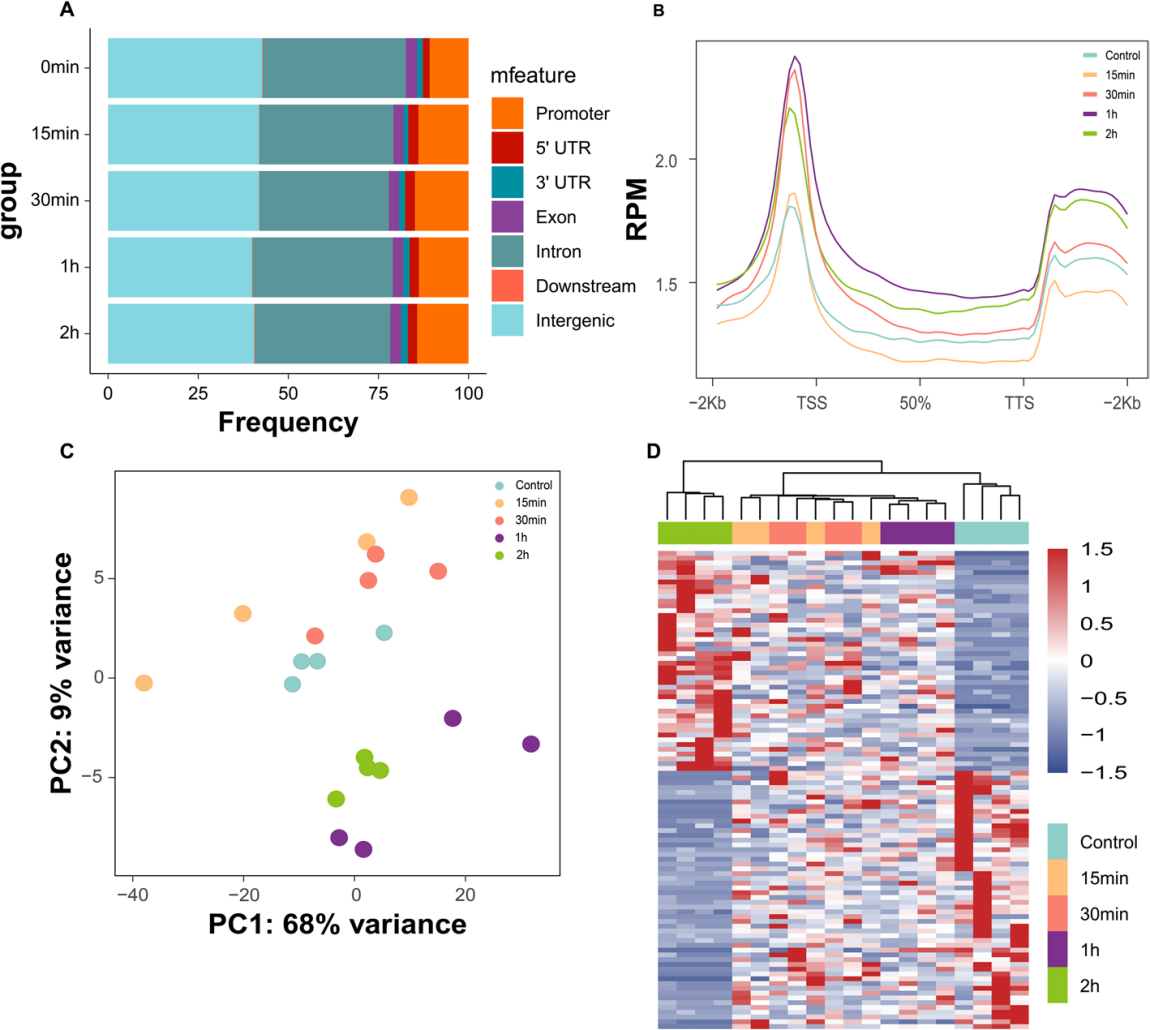
Characterization of KAS-seq distribution at different time points in SMMC-7721 cells treated with sorafenib. **A** Distribution of KAS-seq peaks in the gene coding region across the whole genome in different groups; **B** Distribution of KAS-seq signals at gene-coding regions within the 3000 bp upstream and downstream of the transcription start site (TSS) across different groups; **C** Heatmap showing the distribution of reads in the gene coding regions of KAS-seq samples treated with Sorafenib at different times; **D** Density distribution of peaks at promoter regions across different groups; **E** Density distribution of peaks at exon regions across different groups; **F** Density distribution of peaks at intron regions across different groups

By aligning KAS-seq data to the human reference genome (hg19), we generated a signal distribution profile for single-stranded DNA (ssDNA). At different time points, reads were predominantly enriched in gene coding regions. Among these regions, intron regions occupied the largest proportion, followed by promoter regions (Fig. 2A). Interestingly, the KAS-seq signal intensity varied across gene coding regions at different time points. At the transcription start site (TSS), the KAS-seq signal gradually increased from the control to 15 min, 30 min, and 1 h, and then started to decline from 1 to 2 h (Fig. 2B). Furthermore, we analyzed the distribution density of peaks at the TSS for the control, 15 min, 30 min, 1 h, and 2 h. Notably, the peak density was highest at 1 h (Fig. 2C). Subsequently, we examined the shared and distinct peaks in gene functional regions (promoter, exon, and intron) among the control, 15 min, 30 min, 1-h and 2-h groups. Interestingly, similar peaks were observed across these regions in different groups (Fig. 2D-F). Our findings suggest that the KAS-seq signal is strongest after 1 h of sorafenib treatment and diminishes by 2 h. In addition, PCA and the heatmap using top 100 (*p*-value < 0.01) differentially expressed genes revealed that cell samples could be effectively separated into five subgroups (Figs. S1 A, B).

### Identification and enrichment analysis of differentially expressed genes in early changes

**Fig. 3:**
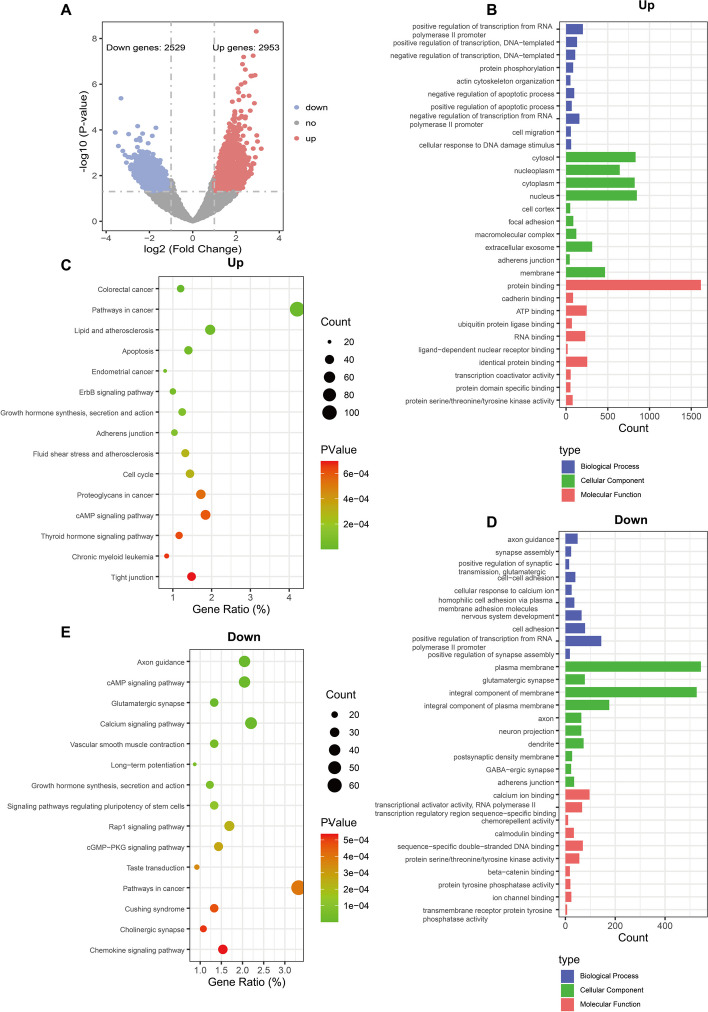
Enrichment analysis of differentially expressed genes in SMMC-7721 cells treated with sorafenib for 1 h. **A** Volcano plot of significantly altered DEGs (|log2 (Fold change) |> 1, *p*-value < 0.05). Upregulated and downregulated DEGs were highlighted respectively in red and blue using SMMC-7721 cells treated with sorafenib for 1 h vs. untreated SMMC-7721 cells. **B** GO enrichment analysis and function exploration of upregulated DEGs. **C** KEGG pathways of upregulated DEGs. **D** GO enrichment analysis of downregulated DEGs. **E** KEGG pathways of downregulated DEGs

Based on the distribution characteristics of KAS-seq signals, we selected the time point (1 h) with the strongest KAS-seq profile signals on gene coding regions to compare with the untreated group (control), for the analysis of genes with early changes (Fig. 2B). Based on the set threshold for significant differences, we finally identified 5482 differentially expressed genes (DEGs), of which 2529 were upregulated and 2953 were downregulated (Fig. 3A). We performed GO and KEGG analyses separately for the upregulated and downregulated genes. Biological process (BP) analysis showed that upregulated DEGs were statistically significantly enriched in positive regulation of transcription from RNA polymerase II promoter and actin cytoskeleton organization, while downregulated DEGs were mainly enriched in positive regulation of transcription from RNA polymerase II promoter and cell adhesion. Cellular component (CC) analysis revealed that upregulated DEGs were mainly enriched in cytosol, nucleus, and downregulated DEGs were mainly enriched in plasma membrane and integral component of membrane. In the molecular function (MF), 1616 upregulated DEGs were enriched in protein binding, whereas downregulated DEGs were mainly enriched in calcium ion binding (Fig. 3B, D). Regarding KEGG, upregulated and downregulated DEGs were both mainly involved in pathways in cancer (Fig. 3C, E).

### Identification and enrichment analysis of efficacy-related differentially expressed genes

**Fig. 4:**
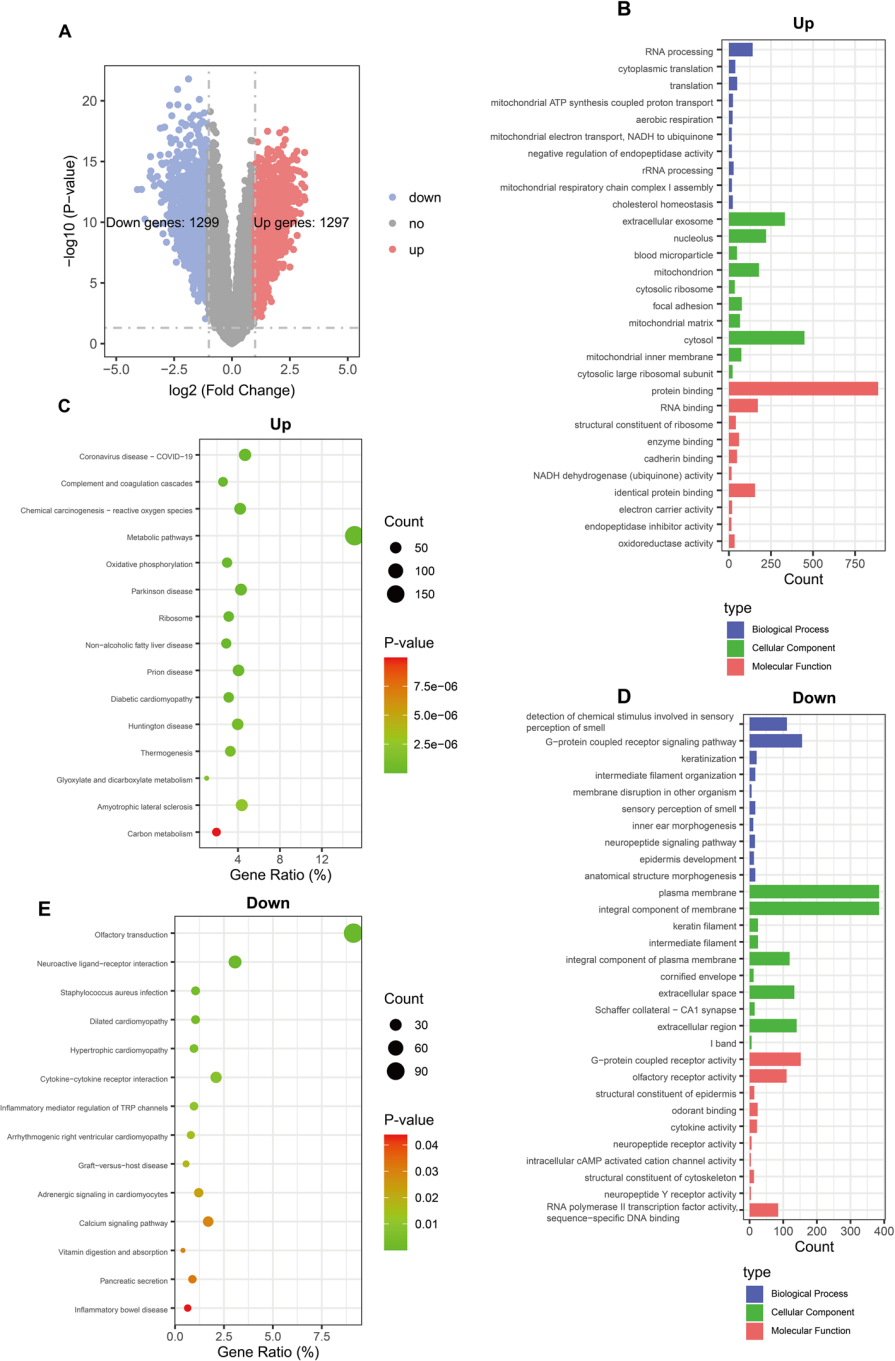
Enrichment analysis of efficacy-related differentially expressed genes (ER-DEGs) in hepatocellular carcinoma patients with ineffective and effective sorafenib treatment (GSE109211). **A** Volcano plot. Significantly altered ERGs (|log2 (Fold change) |> 1, *p*-value < 0.05) were highlighted in red (up) or blue (down) using non-responder vs. responder. **B** GO enrichment analysis and function exploration of upregulated ERGs. **C** KEGG pathways of upregulated ERGs. **D** GO enrichment analysis of downregulated ERGs. **E** KEGG pathways of downregulated ERGs

We compared patients with ineffective and effective sorafenib treatment from the GEO dataset (GSE109211) to identify differentially expressed genes related to therapeutic efficacy (Fig. 1A). We identified a total of 2596 differentially expressed ERGs, of which 1299 genes were upregulated and 1297 were downregulated (Fig. 3A). The GO results showed that upregulated ERGs were mainly involved in biological process (BP) related to RNA processing, cytoplasmic translation and translation, while downregulated ERGs were mainly enriched in detection of chemical stimulus involved in sensory perception of smell and G-protein coupled receptor signaling pathway. Cellular component (CC) analysis revealed that upregulated ERGs were mainly enriched in cytosol and extracellular exosome, and downregulated ERGs were mainly involved in plasma membrane and integral component of membrane. In the molecular function (MF), 887 upregulated ERGs were mainly associated with protein binding, whereas downregulated ERGs were mainly enriched in G-protein coupled receptor activity and olfactory receptor activity (Fig. 4B, D). The results of the KEGG analysis revealed that the metabolic pathways were the main enriched pathways for upregulated ERGs, while olfactory transduction was the main enriched pathway for downregulated ERGs (Fig. 4C, E).

### Selection of hub efficacy-related DEGs

**Fig. 5:**
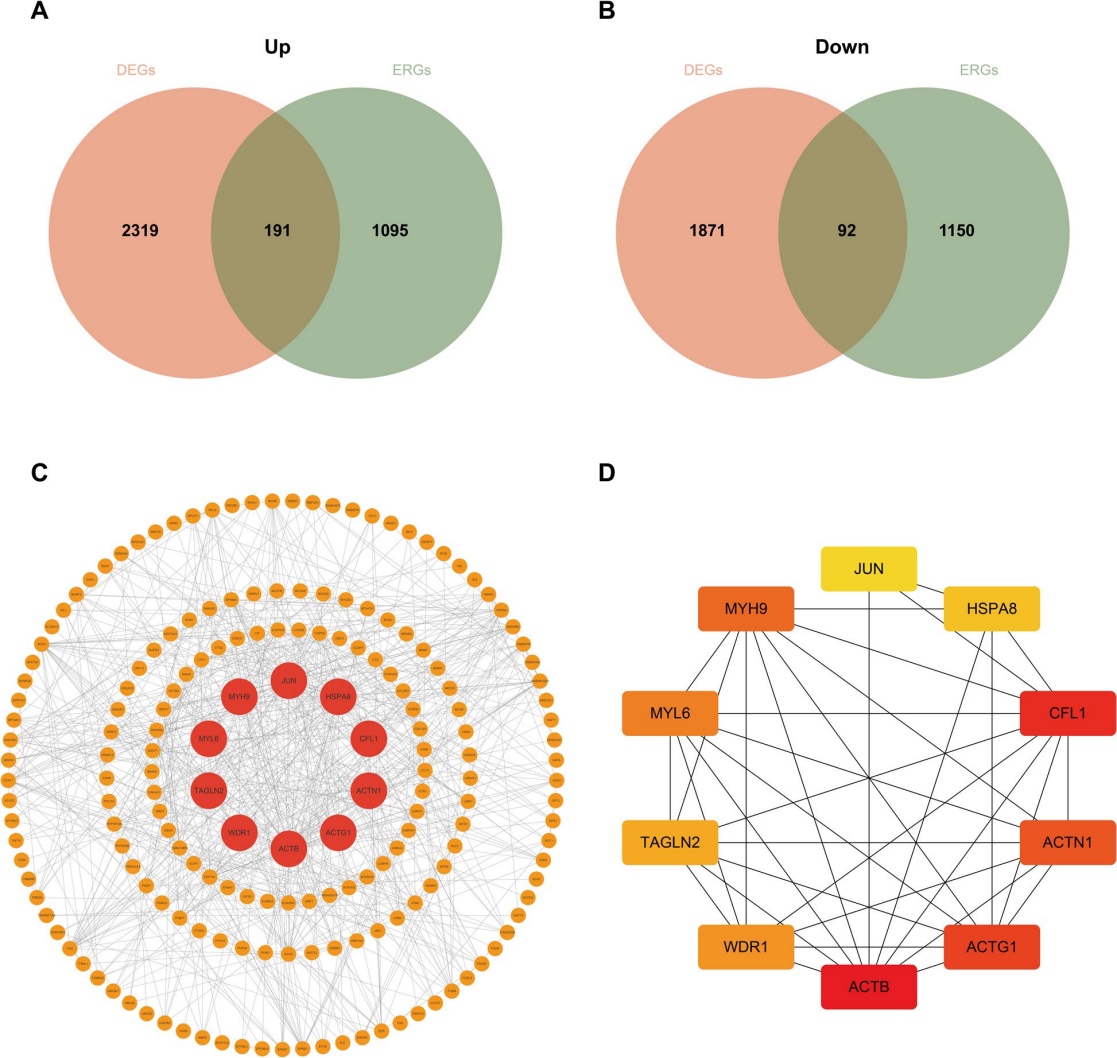
Selection of hub efficacy-related DEGs. **A** Venn plot of the 191 upregulated ER-DEGs. **B** Venn plot of the 92 downregulated ER-DEGs. **C** Protein–protein interaction (PPI) networks of 283 ER-DEGs with confidence score > 0.4. **D** Top 10 hub genes selection performed by the MCC Algorithm

**Fig. 6:**
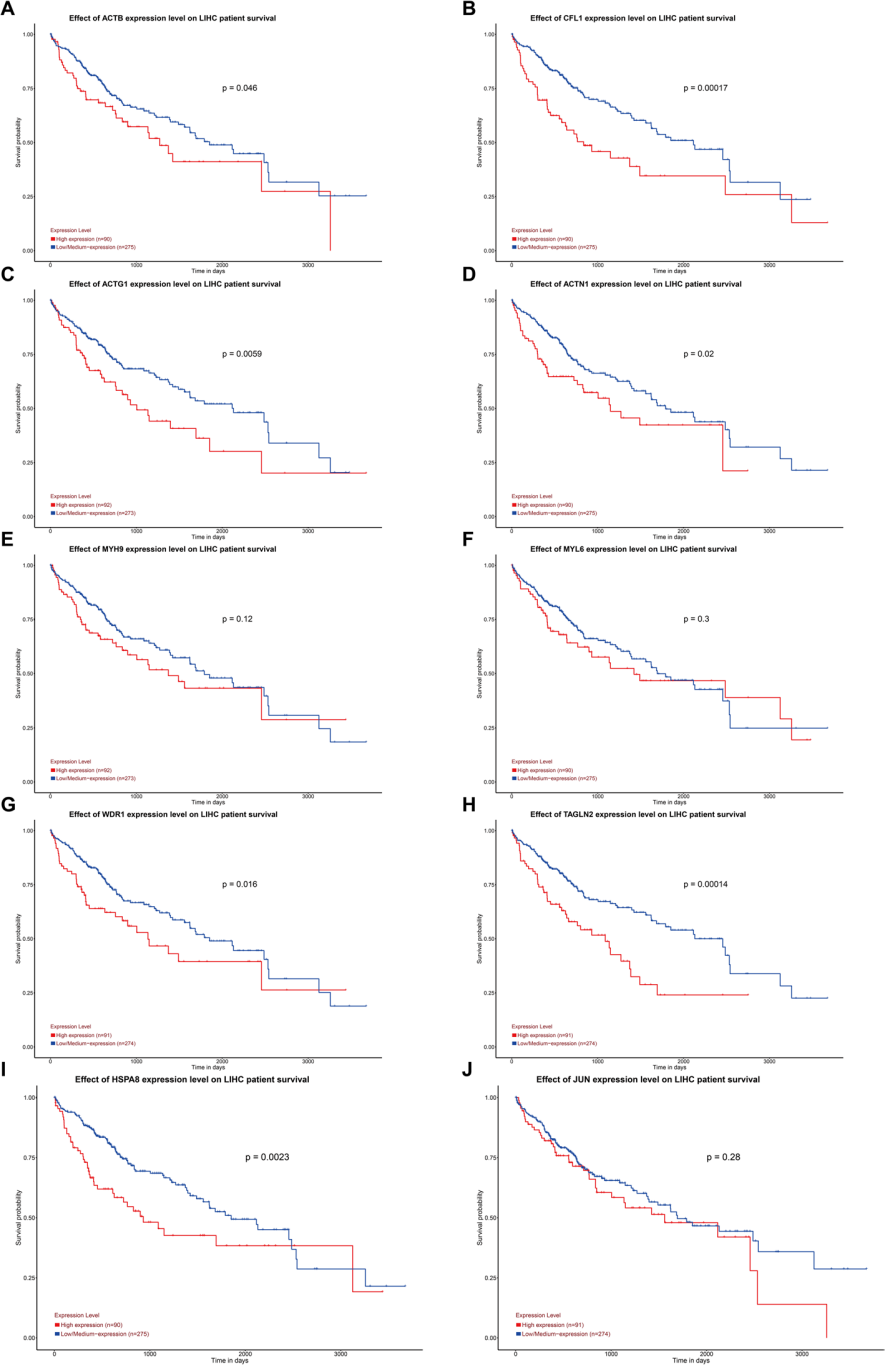
Identification of 10 hub efficacy-related DEGs with prognostic significance using UALCAN. **A-J** The effect of 10 hub efficacy-related DEGs expression level on liver hepatocellular carcinoma (LIHC) patient survival

We intersected the upregulated DEGs and ERGs to obtain 191 genes, and simultaneously intersected the downregulated DEGs and ERGs to obtain 92 genes (Fig. 5A, B). Setting an interaction score > 0.4, the protein–protein interaction (PPI) network was constructed using a total of 283 therapeutic efficacy-related DEGs, and visualized using Cytoscape software (Fig. 5C). To identify the significant genes, we used the maximal clique centrality (MCC) algorithm to calculate the top 10 genes (Fig. 5D). These hub efficacy-related DEGs were ACTB, CFL1, ACTG1, ACTN1, MYH9, MYL6, WDR1, TAGLN2, HSPA8, JUN. Patient survival information for the 10 genes was then plotted using UALCAN database. The results of the survival analysis revealed that elevated expression levels of seven genes are significantly associated with adverse prognosis in patients (Fig. 6A-J).

### Validation of the expression levels of the 7 ERGs

**Fig. 7:**
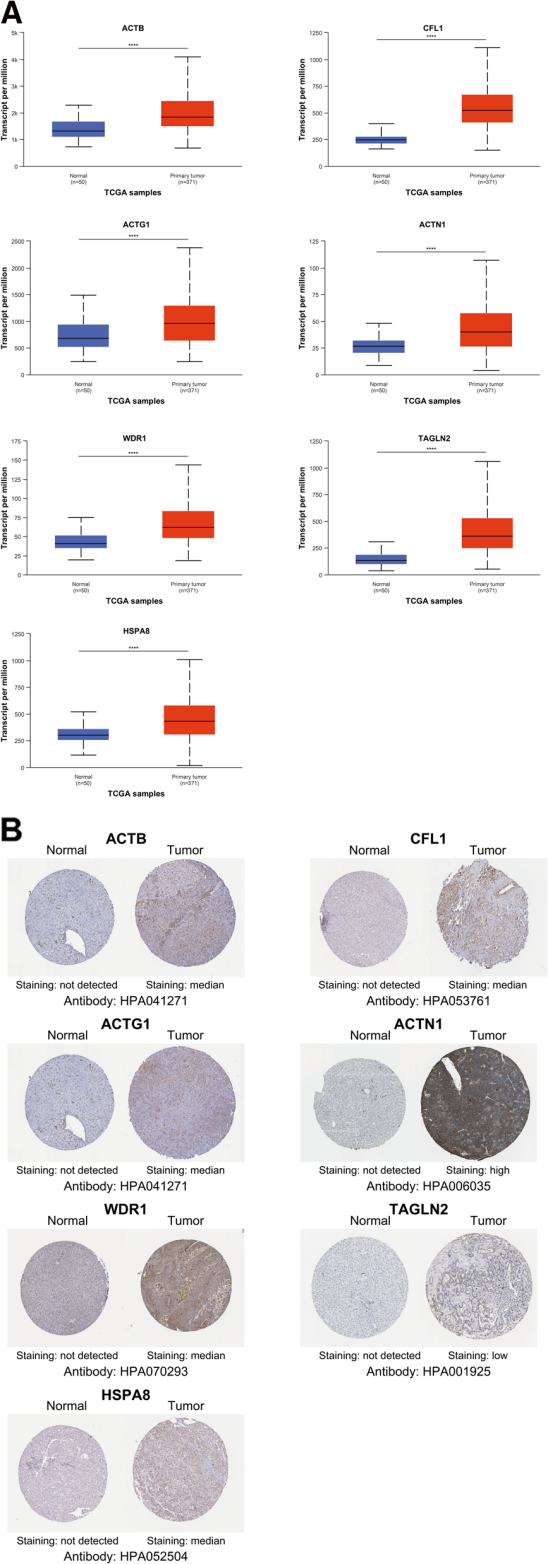
Validation of the expression for 7 hub ERGs. **A** mRNA expression of 7 hub ERGs using were significantly upregulated in patients with LIHC from the UALCAN database (*P < 0.05; **P < 0.01; ***P < 0.001; ****P < 0.0001). **B** Representative immunohistochemistry staining of 7 hub ERGs. Protein expression levels of ACTB, CFL1, ACTG1, ACTN1, WDR1, TAGLN2 and HSPA8 in HCC tissue were obtained from the Human Protein Atlas (HPA)

The transcript expression levels of the 7 hub ERGs were verified using UALCAN database, and the transcript levels of these genes all reached statistical significance. To validate the 7 hub ERGs at the protein level, protein expression data were obtained from Human Protein Atlas (HPA) database. The results of the protein expression levels showed a trend similar to that of the transcript levels (Fig. 7A, B).

### Investigation of statistically significant pathways for the 7 ERGs

**Fig. 8:**
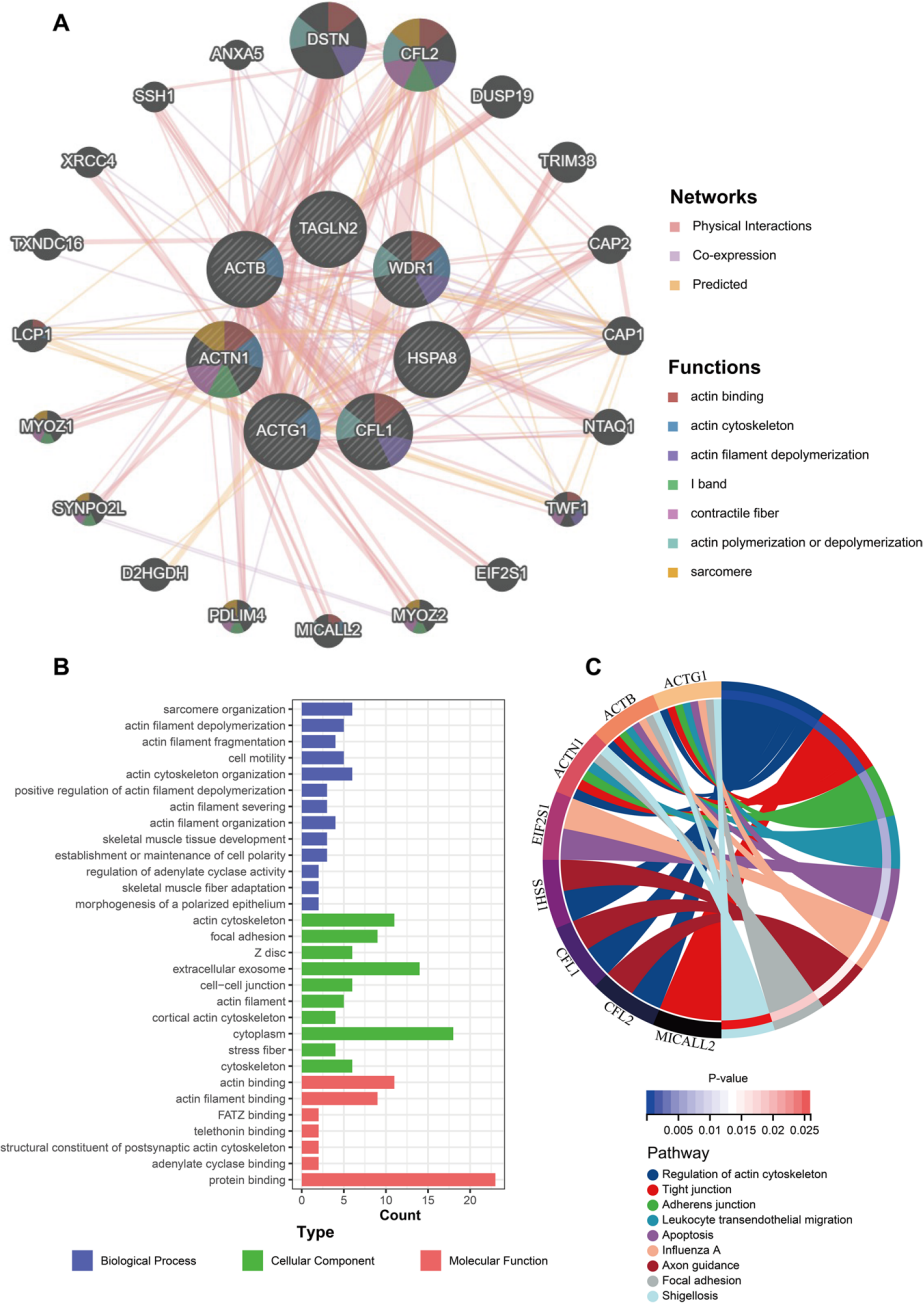
Investigation of statistically significant pathways for the 7 ERGs. **A** The gene–gene interaction network of 7 hub ERGs and 20 neighboring genes was constructed using GeneMANIA. **B** GO enrichment analysis and function exploration of the 27 genes. **C** KEGG pathways of the 27 genes

To study the functions of these 7 ERGs holistically, we constructed a network with 20 neighboring genes using GeneMANIA (Fig. 8A). The result of the network showed that the 27 genes were mainly enriched in actin binding, actin cytoskeleton and actin filament depolymerization, which meant these genes were associated with actin. The GO enrichment analysis showed similar results, with these genes primarily enriched in the actin cytoskeleton organization and sarcomere organization in the Biological Process (BP), mainly enriched in the cytoplasm in the Cellular Component (CC), and primarily enriched in protein binding and actin binding in the Molecular Function (MF) (Fig. 8B). The results of the KEGG analysis showed that these genes were mainly related to the following pathways: regulation of actin cytoskeleton, tight junction, and adherens junction (Fig. 8C).

**Fig. 9:**
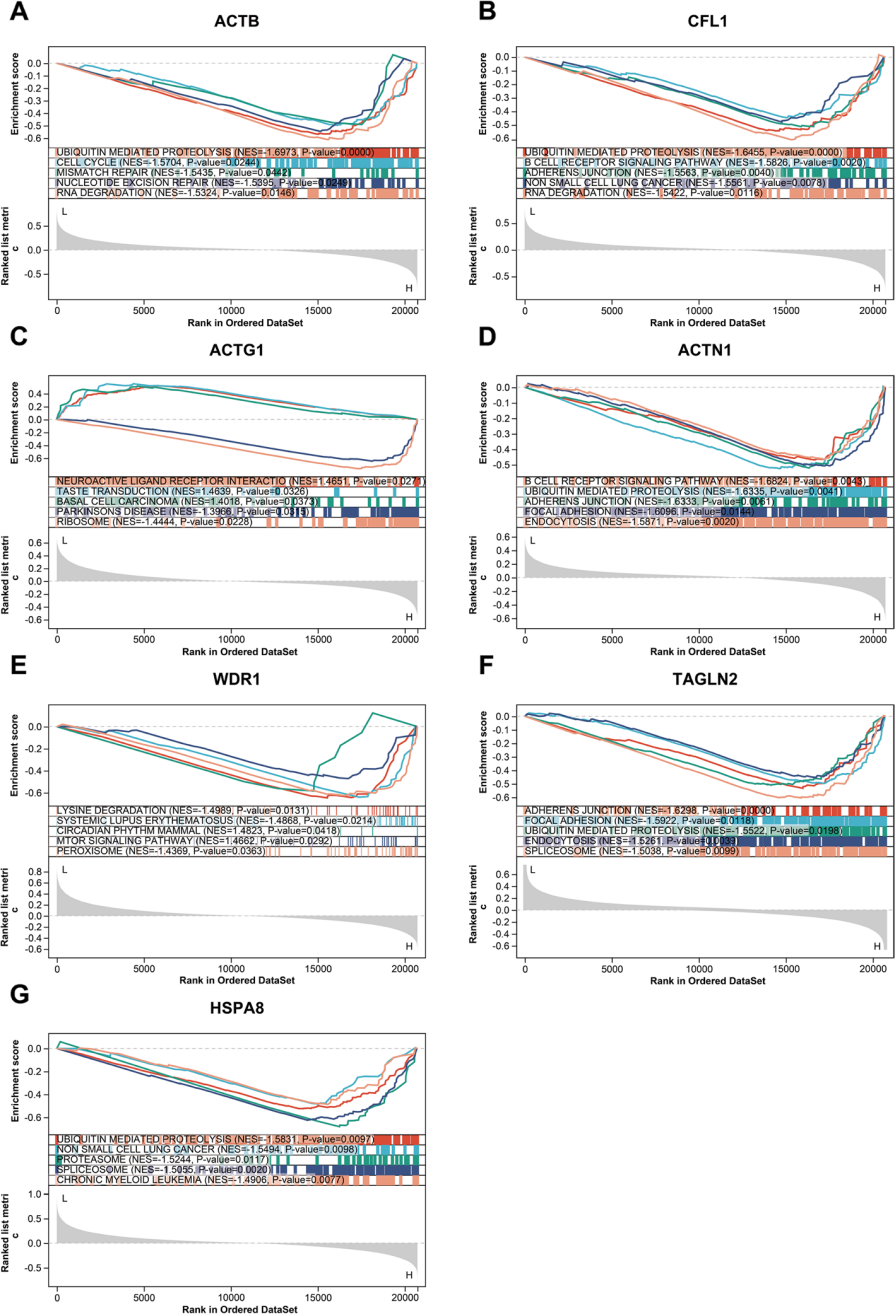
Gene Set Enrichment Analysis (GSEA) was performed using GEO sample data from patients treated solely with sorafenib (GSE109211). **A-G** The gene sets (according to GSEA normalized enrichment score) for ACTB, CFL1, ACTG1, ACTN1, WDR1, TAGLN2, HSPA8. P-value of < 0.05 was considered statistically significant

To further explore the potential functionality of the 7 ERGs, Gene Set Enrichment Analysis (GSEA) was performed using GEO sample data from patients treated solely with sorafenib (GSE109211). As demonstrated in Fig. 9A-G, most genes are enriched in ubiquitin-mediated proteolysis. Additionally, CFL1, ACTN1, and TAGLN2 are enriched in adherens junctions.

## Discussion

Drug-induced stress responses in tumor cells play a crucial role in shaping the ultimate transcriptional patterns leading to drug resistance [42, 43]. Additionally, tumor cells may exhibit transcriptional heterogeneity and leverage the aforementioned stress responses, laying a critical molecular foundation for identifying transcriptional patterns favorable for cancer cell survival [44]. This implies that the transition from sensitive to resistant cells is a gradual adaptation process to drug exposure. Therefore, systematic investigation of drug-treated sensitive cells, observing early-stage gene expression regulatory changes, holds promise for unraveling drug resistance mechanisms from a novel perspective.

In a recent study, researchers from Yale School of Medicine (Katerina A. Politi) and Harvard Medical School (Cigall Kadoch) discovered a correlation between osimertinib resistance and widespread changes in chromatin accessibility. Notably, they demonstrated that the mSWI/SNF complex maintains proliferation and reduces ROS levels in resistant cells [45]. This study provides crucial evidence supporting the involvement of drug-induced stress states in mechanisms of resistance.

In the study, we employed KAS-seq technology to analyze the ssDNA changes in hepatocellular carcinoma cells (SMMC-7721) treated with sorafenib over a two-hour period. Notably, we observed significant differences in KAS-seq signal intensity within gene coding regions at different time points. Specifically, at the transcription start site (TSS), the KAS-seq signal gradually increased from the control to 15 min, 30 min, and 1 h, followed by a decline from 1 to 2 h. Further analysis revealed that the peak density at the TSS was highest at 1 h, suggesting heightened activity of dsDNA during this time. This dynamic response may be triggered by stress reactions in tumor cells.

Based on the KAS-seq signal intensity, we selected the time point corresponding to the highest signal strength (1 h) for differential expression analysis compared to the control group. Subsequently, through functional enrichment, we further confirmed the feasibility of KAS-seq as a sequencing technology for detecting early gene changes in drug-treated cells. In the KEGG enrichment analysis, we observed significant enrichment of DEGs in pathways closely associated with cancer, such as pathways in cancer, MAPK, and cAMP signaling pathways, which also play critical roles in sorafenib resistance. Additionally, we noticed enrichment in pathways closely related to the cell cytoskeleton, including adherens junction, cell cycle, and tight junction. Simultaneously, our analysis revealed a strong association between regulation of transcription from RNA polymerase II promoter and GO biological processes (CC), further confirming that KAS-seq signals primarily originate from transcription reactions involving RNA polymerase II. Furthermore, in the GO molecular function (MF) analysis, most genes within the gene set were associated with protein binding, suggesting the need for further investigation into protein–protein interactions resulting from gene expression changes and their impact on sorafenib resistance following drug treatment.

In the context of clinical cohort studies within molecular evidence-based medicine, which directly reflect patients’ disease status, we gain essential research tools for deciphering disease and pharmacological mechanisms. Furthermore, chemotherapy effectiveness is predominantly limited by drug resistance. Leveraging a clinical cohort treated with sorafenib (GSE109211), we compared patients who responded effectively to sorafenib treatment with those who did not, identifying differentially expressed ERGs [46]. KEGG enrichment analysis revealed that most genes were enriched in metabolic pathways, which are closely associated with previously identified mechanisms of drug resistance. Notably, previous research has highlighted the role of peroxisome proliferator-activated receptor delta (PPARδ) in sorafenib-induced metabolic reprogramming in hepatocellular carcinoma (HCC). PPARδ contributes to enhancing the proliferative capacity and redox homeostasis of sorafenib-resistant HCC cells, while inhibiting PPARδ activity can potentially reverse compensatory metabolic reprogramming in these drug-resistant cells.

To further validate the relevance of early-stage genes identified as sorafenib-mediated changes in hepatocellular carcinoma (HCC) cell resistance, we intersected two sets of differentially expressed genes (DEGs and ERGs). Next, we constructed a protein–protein interaction (PPI) network for the overlapping genes using the STRING database and visualized it using Cytoscape software. Additionally, we applied the Maximum Clique Centrality (MCC) algorithm from the cytoHubba plugin to identify the top 10 key genes within this network. Through survival analysis, we found that the expression levels of 7 genes (ACTB, CFL1, ACTG1, ACTN1, WDR1, TAGLN2, HSPA8) were significantly associated with poor prognosis in patients. High gene expression often leads to worse patient outcomes. These genes were identified through differential expression analysis between patients who were responsive and non-responsive to sorafenib treatment. This suggests that genes potentially related to sorafenib resistance may drive disease progression, further indicating a close relationship between the differentially expressed genes we analyzed and sorafenib resistance. We then validated these findings at both the transcriptional and protein levels.

To further understand the drug resistance mechanisms involved with 7 hub ERGs, we conducted analyses using GeneMANIA, GO, and KEGG, and found that these genes are closely related to the cytoskeleton. According to previous reports, these genes promote the invasiveness and metastasis of tumors, which is often related to changes in the cytoskeleton [47–53]. Previous studies have reported that drug treatment stress can cause molecular and phenotypic changes in tumor cells, i.e., cellular plasticity, thereby inducing tumor drug resistance [24]. This suggests that the remodeling of the cytoskeleton may be related to sorafenib resistance in hepatocellular carcinoma.

Furthermore, when we performed GSEA analysis on these cytoskeleton-related genes using patient samples treated with sorafenib from the GSE109211 dataset, we found that many genes were primarily enriched in ubiquitin-mediated proteolysis. This may suggest their important role in the cytoskeleton.

The research paradigm established in this study holds promise for paving new avenues and research strategies in understanding molecular mechanisms of tumor drug resistance, target discovery, and combination therapies. We hope that this technological breakthrough will extend the lifespans of more late-stage cancer patients.

The study has some limitations. First, the sample size used in the KAS-seq data of this study is relatively small. Second, to identify the most critical genes for subsequent analysis, library sequencing analysis using KAS-seq should be conducted with various hepatocellular carcinoma cells. Additionally, further experiments are required to analyze the mechanisms involved in the early changes of each gene in sorafenib-treated hepatocellular carcinoma cells, as well as the relationship between the genes, the cytoskeleton, and sorafenib resistance in liver cancer cells.

## Conclusion

In summary, based on KAS-seq data, we discovered differential changes in KAS-seq signals at different times after drug treatment of cells. In conjunction with the external dataset (GSE109211), we ultimately found that the cytoskeleton may be closely related to sorafenib resistance in hepatocellular carcinoma. Finally, through GSEA enrichment analysis, we found that ubiquitin-mediated proteolysis may play a key role in the cytoskeleton.

### Supplementary Information

Additional file 1: Fig. S1. The differences between groups at various time points after sorafenib treatment in SMMC-7721 cells. (A) PCA plot of top 100 DEGs with sorafenib treatment at different time points. (B) Heatmap of top 100 DEGs with sample type with sorafenib at different time points.

## Data Availability

Except for GSE109211 from the GEO database, the datasets supporting the conclusions of this article are included within the article. All datasets used and analyzed during the current study are available from the corresponding author on reasonable request.

## Abbreviations

HCC: Hepatocellular carcinoma
TKI: Tyrosine kinase inhibitor
EMT: Epithelial-mesenchymal transition
ssDNA: Single-stranded DNA
dsDNA: Double-stranded DNA
RPA-seq: RPA chromatin immunoprecipitation sequencing
RAD51-seq: RAD51 chromatin immunoprecipitation sequencing
SSDS: Single-stranded DNA sequencing
ChIP-seq: Chromatin immunoprecipitation sequencing
FBS: Fetal bovine serum
GEO: Gene Expression Omnibus
DEGs: Differentially expressed genes
ERGs: Efficacy-related genes
GO: Gene ontology
KEGG: Kyoto Encyclopedia of Genes and Genomes
STRING: Search Tool for the Retrieval of Interacting Genes/Proteins
MCC: Maximal clique centrality
UALCAN: The University of Alabama at Birmingham cancer data analysis
LIHC: Liver hepatocellular carcinoma
HPA: Human Protein Atlas
GSEA: Gene Set Enrichment Analysis
TSS: Transcription start site
TES: Transcription end site
BP: Biological process
CC: Cellular component
MF: Molecular function
OS: Overall survival

## References

[1] Keating GM (2017). Sorafenib: a review in hepatocellular carcinoma. Target Oncol.

[2] Wilhelm SM, Carter C, Tang L, Wilkie D, McNabola A, Rong H, Chen C, Zhang X, Vincent P, McHugh M (2004). BAY 43–9006 exhibits broad spectrum oral antitumor activity and targets the RAF/MEK/ERK pathway and receptor tyrosine kinases involved in tumor progression and angiogenesis. Cancer Res.

[3] Liu L, Cao Y, Chen C, Zhang X, McNabola A, Wilkie D, Wilhelm S, Lynch M, Carter C (2006). Sorafenib blocks the RAF/MEK/ERK pathway, inhibits tumor angiogenesis, and induces tumor cell apoptosis in hepatocellular carcinoma model PLC/PRF/5. Cancer Res.

[4] Prieto-Domínguez N, Ordóñez R, Fernández A, García-Palomo A, Muntané J, González-Gallego J, Mauriz JL (2016). Modulation of autophagy by Sorafenib: effects on treatment response. Front Pharmacol.

[5] Gauthier A, Ho M (2013). Role of sorafenib in the treatment of advanced hepatocellular carcinoma: an update. Hepatol Res.

[6] Rodríguez-Hernández MA, González R, de la Rosa ÁJ, Gallego P, Ordóñez R, Navarro-Villarán E, Contreras L, Rodríguez-Arribas M, González-Gallego J, Álamo-Martínez JM (2018). Molecular characterization of autophagic and apoptotic signaling induced by sorafenib in liver cancer cells. J Cell Physiol.

[7] Ding Z, Pan Y, Shang T, Jiang T, Lin Y, Yang C, Pang S, Cui X, Wang Y, Feng XF (2023). URI alleviates tyrosine kinase inhibitors-induced ferroptosis by reprogramming lipid metabolism in p53 wild-type liver cancers. Nat Commun.

[8] Ping LJCP (2008). Sorafenib plus capecitabine for patients with advanced hepatocellular carcinoma.

[9] Zhai B, Sun XY (2013). Mechanisms of resistance to sorafenib and the corresponding strategies in hepatocellular carcinoma. World J Hepatol.

[10] Lackner MR, Wilson TR, Settleman J (2012). Mechanisms of acquired resistance to targeted cancer therapies. Future Oncol.

[11] Bagrodia S, Smeal T, Abraham RT (2012). Mechanisms of intrinsic and acquired resistance to kinase-targeted therapies. Pigment Cell Melanoma Res.

[12] Bottsford-Miller JN, Coleman RL, Sood AK (2012). Resistance and escape from antiangiogenesis therapy: clinical implications and future strategies. J Clin Oncol.

[13] Makol A, Kaur H, Sharma S, Kanthaje S, Kaur R, Chakraborti A (2020). Vimentin as a potential therapeutic target in sorafenib resistant HepG2, a HCC model cell line. Clin Mol Hepatol.

[14] Debaugnies M, Rodríguez-Acebes S, Blondeau J, Parent MA, Zocco M, Song Y, de Maertelaer V, Moers V, Latil M, Dubois C (2023). RHOJ controls EMT-associated resistance to chemotherapy. Nature.

[15] Zhao X, He R, Liu Y, Wu Y, Kang L (2017). UPregulated single-stranded DNA-binding protein 1 induces cell chemoresistance to cisplatin in lung cancer cell lines. Mol Cell Biochem.

[16] Bélanger F, Fortier E, Dubé M, Lemay JF, Buisson R, Masson JY, Elsherbiny A, Costantino S, Carmona E, Mes-Masson AM (2018). Replication Protein A availability during DNA replication stress is a major determinant of cisplatin resistance in ovarian cancer cells. Cancer Res.

[17] Patel A, Seraia E, Ebner D, Ryan AJ (2020). Adefovir dipivoxil induces DNA replication stress and augments ATR inhibitor-related cytotoxicity. Int J Cancer.

[18] Mertz TM, Collins CD, Dennis M, Coxon M, Roberts SA (2022). APOBEC-Induced mutagenesis in cancer. Annu Rev Genet.

[19] Chen FX, Smith ER, Shilatifard A (2018). Born to run: control of transcription elongation by RNA polymerase II. Nat Rev Mol Cell Biol.

[20] Bell SP, Dutta A (2002). DNA replication in eukaryotic cells. Annu Rev Biochem.

[21] Hustedt N, Durocher D (2016). The control of DNA repair by the cell cycle. Nat Cell Biol.

[22] Li X, Heyer WD (2008). Homologous recombination in DNA repair and DNA damage tolerance. Cell Res.

[23] Ginno PA, Lott PL, Christensen HC, Korf I, Chédin F (2012). R-loop formation is a distinctive characteristic of unmethylated human CpG island promoters. Mol Cell.

[24] Yuan S, Norgard RJ, Stanger BZ (2019). Cellular Plasticity in Cancer. Cancer Discov.

[25] Yamane A, Robbiani DF, Resch W, Bothmer A, Nakahashi H, Oliveira T, Rommel PC, Brown EJ, Nussenzweig A, Nussenzweig MC, Casellas R (2013). RPA accumulation during class switch recombination represents 5'-3' DNA-end resection during the S-G2/M phase of the cell cycle. Cell Rep.

[26] Lange J, Yamada S, Tischfield SE, Pan J, Kim S, Zhu X, Socci ND, Jasin M, Keeney S (2016). The landscape of mouse meiotic double-strand break formation, processing, and repair. Cell.

[27] Paiano J, Wu W, Yamada S, Sciascia N, Callen E, Paola Cotrim A, Deshpande RA, Maman Y, Day A, Paull TT, Nussenzweig A (2020). ATM and PRDM9 regulate SPO11-bound recombination intermediates during meiosis. Nat Commun.

[28] Hinch AG, Becker PW, Li T, Moralli D, Zhang G, Bycroft C, Green C, Keeney S, Shi Q, Davies B, Donnelly P (2020). The configuration of RPA, RAD51, and DMC1 binding in meiosis eeveals the nature of critical recombination intermediates. Mol Cell.

[29] Khil PP, Smagulova F, Brick KM, Camerini-Otero RD, Petukhova GV (2012). Sensitive mapping of recombination hotspots using sequencing-based detection of ssDNA. Genome Res.

[30] Zhou ZX, Zhang MJ, Peng X, Takayama Y, Xu XY, Huang LZ, Du LL (2013). Mapping genomic hotspots of DNA damage by a single-strand-DNA-compatible and strand-specific ChIP-seq method. Genome Res.

[31] Wu T, Lyu R, You Q, He C (2020). Kethoxal-assisted single-stranded DNA sequencing captures global transcription dynamics and enhancer activity in situ. Nat Methods.

[32] Lyu R, Wu T, Zhu AC, West-Szymanski DC, Weng X, Chen M, He C (2022). KAS-seq: genome-wide sequencing of single-stranded DNA by N(3)-kethoxal-assisted labeling. Nat Protoc.

[33] Hafner M, Niepel M, Chung M, Sorger PK (2016). Growth rate inhibition metrics correct for confounders in measuring sensitivity to cancer drugs. Nat Methods.

[34] Clark NA, Hafner M, Kouril M, Williams EH, Muhlich JL, Pilarczyk M, Niepel M, Sorger PK, Medvedovic M (2017). GRcalculator: an online tool for calculating and mining dose-response data. BMC Cancer.

[35] Warde-Farley D, Donaldson SL, Comes O, Zuberi K, Badrawi R, Chao P, Franz M, Grouios C, Kazi F, Lopes CT (2010). The GeneMANIA prediction server: biological network integration for gene prioritization and predicting gene function. Nucleic Acids Res.

[36] da Huang W, Sherman BT, Lempicki RA (2009). Systematic and integrative analysis of large gene lists using DAVID bioinformatics resources. Nat Protoc.

[37] Szklarczyk D, Kirsch R, Koutrouli M, Nastou K, Mehryary F, Hachilif R, Gable AL, Fang T, Doncheva NT, Pyysalo S (2023). The STRING database in 2023: protein-protein association networks and functional enrichment analyses for any sequenced genome of interest. Nucleic Acids Res.

[38] Shannon P, Markiel A, Ozier O, Baliga NS, Wang JT, Ramage D, Amin N, Schwikowski B, Ideker T (2003). Cytoscape: a software environment for integrated models of biomolecular interaction networks. Genome Res.

[39] Chandrashekar DS, Karthikeyan SK, Korla PK, Patel H, Shovon AR, Athar M, Netto GJ, Qin ZS, Kumar S, Manne U (2022). UALCAN: an update to the integrated cancer data analysis platform. Neoplasia.

[40] Subramanian A, Tamayo P, Mootha VK, Mukherjee S, Ebert BL, Gillette MA, Paulovich A, Pomeroy SL, Golub TR, Lander ES, Mesirov JP (2005). Gene set enrichment analysis: a knowledge-based approach for interpreting genome-wide expression profiles. Proc Natl Acad Sci USA.

[41] Liberzon A, Subramanian A, Pinchback R, Thorvaldsdóttir H, Tamayo P, Mesirov JP (2011). Molecular signatures database (MSigDB) 3.0. Bioinformatics.

[42] Sesumi Y, Suda K, Mizuuchi H, Kobayashi Y, Sato K, Chiba M, Shimoji M, Tomizawa K, Takemoto T, Mitsudomi T (2017). Effect of dasatinib on EMT-mediated-mechanism of resistance against EGFR inhibitors in lung cancer cells. Lung Cancer.

[43] Ashrafizadeh M, Mirzaei S, Hashemi F, Zarrabi A, Zabolian A, Saleki H, Sharifzadeh SO, Soleymani L, Daneshi S, Hushmandi K (2021). New insight towards development of paclitaxel and docetaxel resistance in cancer cells: EMT as a novel molecular mechanism and therapeutic possibilities. Biomed Pharmacother.

[44] Pérez-Velázquez J, Rejniak KA (2020). Drug-induced resistance in micrometastases: analysis of spatio-temporal cell lineages. Front Physiol.

[45] de Miguel FJ, Gentile C, Feng WW, Silva SJ, Sankar A, Exposito F, Cai WL, Melnick MA, Robles-Oteiza C, Hinkley MM (2023). Mammalian SWI/SNF chromatin remodeling complexes promote tyrosine kinase inhibitor resistance in EGFR-mutant lung cancer. Cancer Cell.

[46] Shankaraiah N, Nekkanti S, Ommi O (2019). P SL: Diverse targeted approaches to battle multidrug resistance in cancer. Curr Med Chem.

[47] Guo C, Liu S, Wang J, Sun MZ, Greenaway FT (2013). ACTB in cancer. Clin Chim Acta.

[48] Yan Y, Xu H, Zhang L, Zhou X, Qian X, Zhou J, Huang Y, Ge W, Wang W (2019). RRAD suppresses the Warburg effect by downregulating ACTG1 in hepatocellular carcinoma. Onco Targets Ther.

[49] Chen Q, Zhou XW, Zhang AJ, He K (2021). ACTN1 supports tumor growth by inhibiting Hippo signaling in hepatocellular carcinoma. J Exp Clin Cancer Res.

[50] Ji C, Zhao J, Chen H, Wang Z, Cai T, Tian C, Wang C, Liu D, Ye B, Fu M (2023). Single-cell RNA sequencing reveals the lineage of malignant epithelial cells and upregulation of TAGLN2 promotes peritoneal metastasis in gastric cancer. Clin Transl Oncol.

[51] An R, Wang J, Chen X, Xu R, Hu J, Liu Z, Wei C, Zhang C, Yuan B (2022). YAP signaling is involved in WDR1-regulated proliferation and migration of non-small-cell lung cancer cells. Exp Biol Med (Maywood).

[52] Wang Y, Zhao M, Zhao L, Geng Y, Li G, Chen L, Yu J, Yuan H, Zhang H, Yun H (2023). HBx-Induced HSPA8 Stimulates HBV Replication and Suppresses Ferroptosis to support liver cancer progression. Cancer Res.

[53] Zhang L, Chai Z, Kong S, Feng J, Wu M, Tan J, Yuan M, Chen G, Li Z, Zhou H (2021). Nujiangexanthone A Inhibits Hepatocellular Carcinoma Metastasis via Down Regulation of Cofilin 1. Front Cell Dev Biol.

