# Supplementary figures and images for: Cytoskeletal gene alterations linked to sorafenib resistance in hepatocellular carcinoma
Source: World J Surg Oncol. 2024 Jun 7;22:152. doi: 10.1186/s12957-024-03417-2 (PMC11157844; doi:10.1186/s12957-024-03417-2)

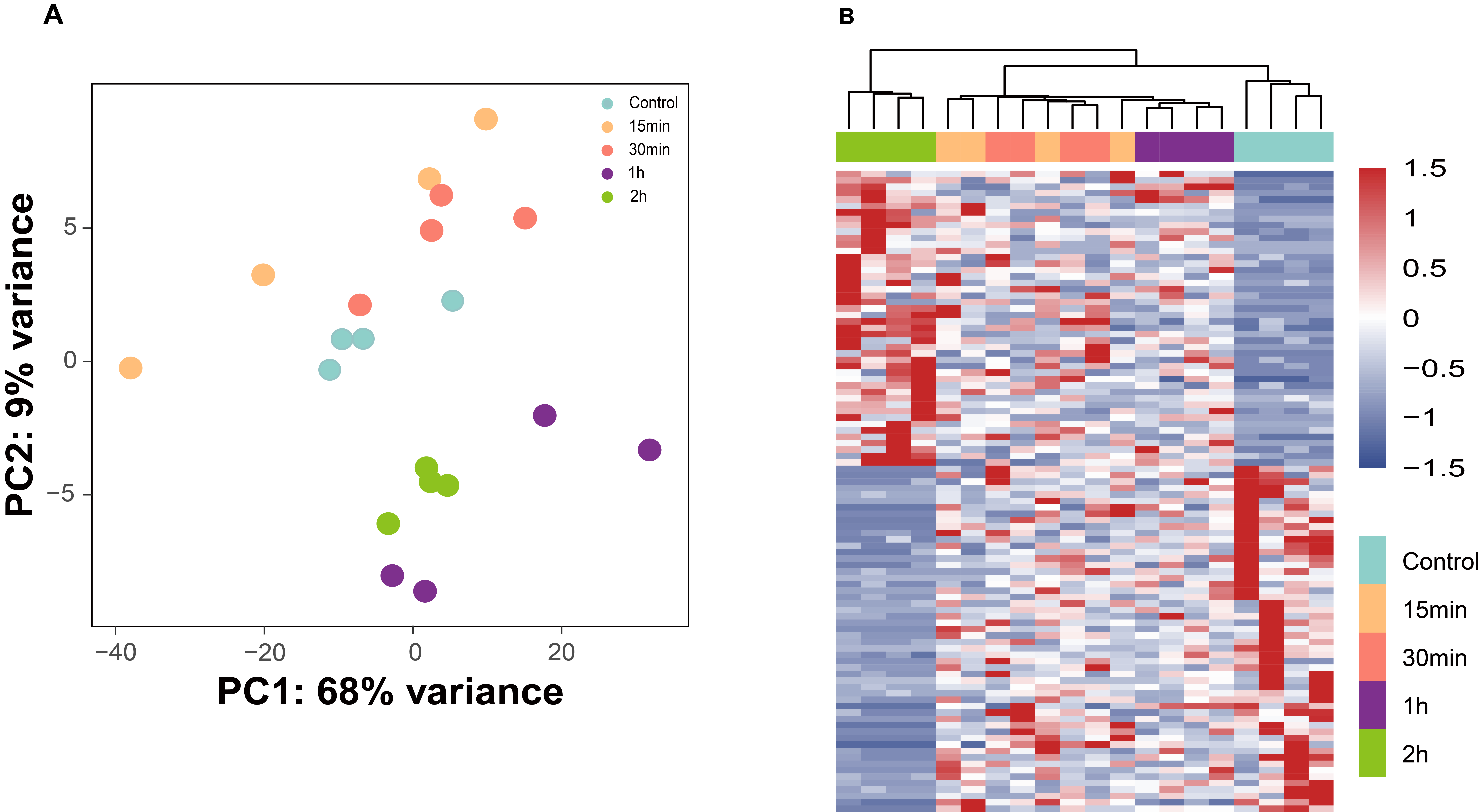

Supplement: Supplementary file 1 — Additional file 1: Fig. S1. The differences between groups at various time points after sorafenib treatment in SMMC-7721 cells. (A) PCA plot of top 100 DEGs with sorafenib treatment at different time points. (B) Heatmap of top 100 DEGs with sample type with sorafenib at different time points. [file 12957_2024_3417_MOESM1_ESM.png]
